# A Regulator Based “Semi-Targeted” Approach to Activate Silent Biosynthetic Gene Clusters

**DOI:** 10.3390/ijms22147567

**Published:** 2021-07-15

**Authors:** Erik Mingyar, Lucas Mühling, Andreas Kulik, Anika Winkler, Daniel Wibberg, Jörn Kalinowski, Kai Blin, Tilmann Weber, Wolfgang Wohlleben, Evi Stegmann

**Affiliations:** 1 Department of Microbiology and Biotechnology, Interfaculty Institute of Microbiology and Infection Medicine, University of Tübingen Auf der Morgenstelle 28, 72076 Tübingen, Germany; erik.mingyar@uni-tuebingen.de (E.M.); lucas.muehling96@gmail.com (L.M.); andreas.kulik@uni-tuebingen.de (A.K.); wolfgang.wohlleben@biotech.uni-tuebingen.de (W.W.); 2 German Center for Infection Research (DZIF), Partner Site Tübingen, 72076 Tübingen, Germany; 3 Department of Microbial Bioactive Compounds, Interfaculty Institute of Microbiology and Infection Medicine, University of Tübingen, Auf der Morgenstelle 28, 72076 Tübingen, Germany; 4 Center for Biotechnology (CeBiTec), Universität Bielefeld, 33615 Bielefeld, Germany; awinkler@cebitec.uni-bielefeld.de (A.W.); dwibberg@cebitec.uni-bielefeld.de (D.W.); joern@cebitec.uni-bielefeld.de (J.K.); 5 Novo Nordisk Foundation Center for Biosustainability, Technical University of Denmark, Kemitorvet, Building 220, 2800 Kgs. Lyngby, Denmark; kblin@biosustain.dtu.dk (K.B.); tiwe@biosustain.dtu.dk (T.W.); 6 Cluster of Excellence EXC 2124—Controlling Microbes to Fight Infections, 72076 Tübingen, Germany

**Keywords:** regulation, streptomyces, secondary metabolites, silent biosynthetic gene cluster

## Abstract

By culturing microorganisms under standard laboratory conditions, most biosynthetic gene clusters (BGCs) are not expressed, and thus, the products are not produced. To explore this biosynthetic potential, we developed a novel “semi-targeted” approach focusing on activating “silent” BGCs by concurrently introducing a group of regulator genes into streptomycetes of the Tübingen strain collection. We constructed integrative plasmids containing two classes of regulatory genes under the control of the constitutive promoter ermE*p (cluster situated regulators (CSR) and *Streptomyces* antibiotic regulatory proteins (SARPs)). These plasmids were introduced into *Streptomyces sp.* TÜ17, *Streptomyces sp*. TÜ10 and *Streptomyces sp*. TÜ102. Introduction of the CSRs-plasmid into strain *S. sp*. TÜ17 activated the production of mayamycin A. By using the individual regulator genes, we proved that Aur1P, was responsible for the activation. In strain *S. sp*. TÜ102, the introduction of the SARP-plasmid triggered the production of a chartreusin-like compound. Insertion of the CSRs-plasmid into strain *S. sp*. TÜ10 resulted in activating the warkmycin-BGC. In both recombinants, activation of the BGCs was only possible through the simultaneous expression of *aur1PR3* and *griR* in *S. sp*. TÜ102 and *aur1P* and *pntR* in of *S. sp*. TÜ10.

## 1. Introduction 

Actinomycetes produce more than two-thirds of the antibiotics used in medicine and agriculture, as well as numerous antitumour agents, and several eukaryotic cell differentiation effectors, such as apoptosis inducers and inhibitors [1]. To date (10 June 2021), more than two thousand genomes of filamentous actinomycetes have been sequenced and annotated (https://jgi.doe.gov/) [2]. Their analysis using genome mining tools like anti-SMASH [3,4,5,6,7,8], PRISM [9] or NaPDoS [10] revealed that each producer has a much higher potential to synthesise natural products than was assumed for many years. This assumption was because strains cultivated under standard laboratory conditions produced only a limited number of compounds. 

Thus, to activate the so-called “silent” biosynthetic gene clusters (BGCs) and to improve their biosynthetic potential various strategies have been applied, including culture media modifications, chemical or antibiotic treatments [11,12], heterologous gene expression in different hosts, or co-culture with cohabiting microbes [13,14].

Although, in several cases, these approaches led to activating silent clusters, these are non-targeted methods whose success is unpredictable. A more targeted approach is the manipulation of the genes encoding cluster-situated regulators (CSRs) [12]. CSRs directly address the biosynthesis of antibiotics by responding to pleiotropic [15] or global regulators [16,17,18] and subsequently regulating the expression of the biosynthetic genes within the cognate clusters [19]. However, not all BGCs are regulated according to the same principles. Some BGCs include multiple CSRs, e.g., in the jadomycin BGC [20], whereas others, such as the sansanmycin BGC, comprise only one CSR [12,19]. Manipulation of the CSRs has a major impact on the production levels of the corresponding antibiotic; overexpression of CSR activators or the deletion of CSR repressors lead to activating numerous BGCs encoding natural products, such as nikkomycin [21] in *S. ansochromogenes*, polyoxin [22] in *S. cacaoi*, stambomycin in *S. ambofaciens* [23], jadomycin B in *S. venezuelae* [24] or ristomycin in *A. japonicum* [25]. 

CSRs are classified into different protein families based on sequence or structural similarities [26,27,28]. The most common and best-described regulators belong to the LuxR, MarR, Spo0J-ParB, TetR, or ARR protein families and are known to control BGC transcription [28]. A particular role in activating BGCs in streptomycetes is played by the *Streptomyces* antibiotic regulatory protein (SARP) family regulators which are well-known activators of antibiotic biosynthesis. 

The LuxR protein from *Vibrio fischeri*, a transcription activator for the quorum-sensitive control of luminescence, is a prototype of a large protein family of regulators. These regulators typically consist of less than 250 amino acids [29]. They are characterised by a C-terminal DNA-binding helix turn helix (HTH) and an N-terminal domain that responds to chemical signalling molecules, such as γ-butyrolactones [28,30]. LuxR proteins positively regulate the biosynthesis of secondary metabolites, such as the PimM regulator of the pimaricin BGC in *Streptomyces natalensis* [31] or FkbN of the tacrolimus BGC in *Streptomyces tsukubaensis* [32]. By comparing the promoter regions of more than ten genes regulated by FkbN, the binding motif of LuxR-like proteins (GGNNNNCCC) could be defined [33]. 

Members of the MarR protein family (multiple antibiotic resistance regulator family) bind to palindromic regions [34] and initiate the transcription of BGCs. As an example, the MarR-type regulator PntR activates the expression of the pentalenolactone BGC in *Streptomyces arenae* TÜ469 [35]. A similar BGC is activated in *Streptomyces exfoliates* by the PenR regulator. The SAV_2989 gene product regulates positively the biosynthesis of neopentalenolactone in *Streptomyces avermitilis*. Interestingly, all three regulators bind to 37 bp consensus sequences harbouring two highly conserved motifs GAAAT(A/G)(T/C)ATCG and CTTAT(A/G)TA(A/G)GCT [35]. 

The first complete regulatory pathway has been described for StrR (streptomycin biosynthesis operon regulator), controlling the biosynthesis of the aminoglycoside antibiotic streptomycin. StrR is a member of the AdpA regulon, with AdpA, in turn, being a central transcriptional regulator in the A-factor regulatory cascade that leads to morphological development and secondary metabolism in *Streptomyces griseus* [15]. The A-factor, a gamma-butyrolactone biosynthesised by AfsA, gradually accumulates in a growth-dependent manner. It induces the transcription of *adpA*, encoding a transcriptional activator. For this purpose, it binds to the A-factor receptor protein (ArpA), which is bound to the *adpA* promoter and causes its dissociation. AdpA subsequently activates a number of genes with various functions required for morphological development and secondary metabolism, thus forming the AdpA regulon [15,36].

The presence of A-factor homologues in a wide variety of *Streptomyces* species and distantly related bacteria implies that gamma-butyrolactones are widespread cellular signalling molecules in microorganisms [36,37]. StrR-binding sites presenting the consensus sequence GTTCGActG(N)_11_CagTcGAAc were identified in the streptomycin gene clusters of *S. griseus* and *S. glaucescens* [11,38]. 

A frequently observed regulatory protein family in *Streptomyces* are the SARPs [39]. SARPs are cluster-situated activators mostly found within BGCs encoding the biosynthesis of antibiotics. The first studied and characterised SARP regulator was ActII-ORF4 controlling the actinorhodin (Act) biosynthesis in *S. coelicolor* [40]. SARPs include a N-terminal winged HTH motif, which is crucial for binding to a conserved DNA sequence. The binding motif of SARPs consists of heptameric repeats with 4 bp spacers located stringently 8 bp upstream of −10 promoter region [41].

Additional regulators commonly present in *Streptomyces* that influence antibiotic production are two-component systems (TCS) consisting of a sensor histidine kinase and a response regulator. TCSs are also essential e.g., in communication or in adaptation to the environment. The sensor histidine kinase senses a specific signal from the environment (ligand). Upon ligand binding, it gets autophosphorylated and subsequently transfers the phosphoryl group to the cognate response regulator, which then activates or represses the transcription of target genes [28]. AbsA1/AbsA2 is an extensively studied TCS of *S. coelicolor*, which is involved in activating calcium-dependent antibiotic (CDA) transcription [42]. 

On the other hand, atypical response regulators also exist (ARR). They display homologies to the response regulators of TCS, but lack the important residues for phosphorylation, which are essential for signal transmission in TCS. Accordingly, ARRs use a different, not fully understood signal response mechanism [43]. Aur1P, the activator of the angucycline-like antibiotic auricin, is a member of ARRs family regulators. The direct binding of Aur1P to the auricin promoter region regulates the transcription of auricin biosynthesis. DNase-I footprint experiments revealed that the Aur1P binding sequence is a tandem repeat sequence TCCCTTG separated by a 24 bp spacing region accompanied by sequence regions CCTTG and CCT [44,45].

Although the regulators can be classified according to their sequences, in most cases, it is not possible to deduce from this neither their function as activator or repressor, nor the type of product of the respective BGC they are regulating [46]. 

In our “semi-targeted” approach we used the activating nature of regulators to initiate the transcription of silent clusters. For this purpose, representative CSR genes of the protein families LuxR, StrR, ARR, MarR, SARPs were introduced into different strains of the Tübingen strain collection. We demonstrated the development of a very promising method that circumvents the limitations of classical regulatory approaches to discover new natural compounds.

## 2. Results and Discussion

### 2.1. Selection and Cloning of Regulatory Genes for Activation of Silent Clusters

To exploit the full biosynthetic potential of actinomycetes, a variety of methods to active silent clusters has been developed in many scientific groups worldwide. These include, on the one hand, methods for untargeted biosynthetic gene cluster (BGC) activation, such as the introduction of pleiotropic activators [47], the use of chemical elicitors [48] or transcription factor decoys [49]. Thus, activating silent clusters is more of a random event. On the other side, targeted approaches need specialised constructs for each BGC of interest, i.e., methods, such as promoter refactoring or manipulation of pathway-specific transcriptional regulators [50,51]. This procedure requires specific knowledge of the BGC and is time-consuming.

**Figure 1:**
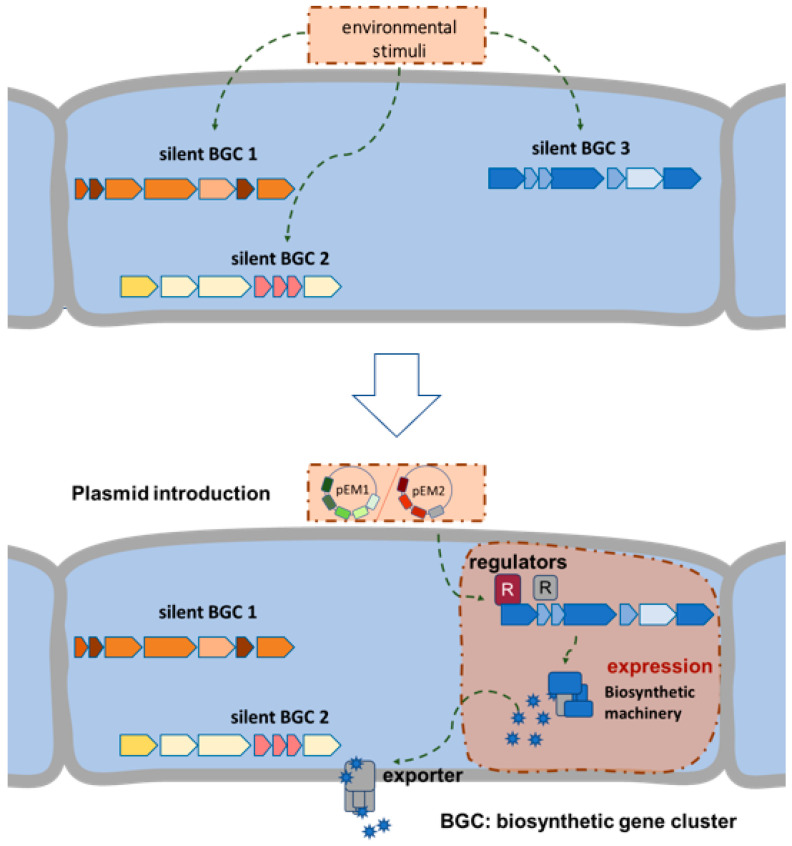
Scheme of the “semi-targeted” approach to activate silent BGCs in *Actinomyces*. pEM1, plasmid carrying different cluster situated regulator genes (CSRs); pEM2, plasmid carrying genes encoding different *Streptomyces* antibiotic regulatory proteins (SARPs).

We chose a “semi-targeted” approach that takes an intermediate position between the untargeted and targeted one (Figure 1). Our work is focusing on activating silent clusters by introducing heterologous regulatory genes in strains of the Tübingen strain collection, which have previously been evaluated for their biosynthetic potential. We reasoned that the production of heterologous cluster situated regulators (CSRs) from various protein families would enable the detection of metabolites that would otherwise not be produced. It is feasible that the binding of these regulators to the promoter region of genes encoding the biosynthesis of bioactive compounds could activate their transcription without prior knowledge of the specific BGC sequences and by overcoming the necessity of an environmental signal.

First, we selected CSRs regulator genes from various BGCs, which were experimentally characterised and validated. The genes were cloned as a single operon under the control of the strong constitutive promoter ermEp* into the integrative vector pRM4 [52]. Two plasmids (pEM1 and pEM2) were constructed by a classical cloning approach. These plasmids are carrying different sets of regulatory genes amplified of the genes from the respective *Streptomyces* strains (Table 1). 

Plasmid pEM1 contained four different genes *aur1P—pntR—strR—fkbN*. Particular focus was taken on including one representative from each type of CSR (Table 1). 

Plasmid pEM2 carries five different *Streptomyces* antibiotic regulatory protein (SARP) genes, *actIIORF4—red—griR—aur1PR3—papR2* (Table 1). Our aim was to apply SARPs, which are highly distinctive, and which regulate the biosynthesis of structurally diverse compounds. Based on all small SARPs available in the NCBI database, five SARPs were selected, which regulate the synthesis of structurally different products. Using phylogenetic analyses, we confirmed that the regulators do not display any homologies to each other (SI. Figure S1A, SI. Figure S1B).

### 2.2. Activation of Silent BGCs in Strains of the Tübingen Strain Collection 

Three strains isolated from soil in Switzerland, *Streptomyces sp.* TÜ17, *Streptomyces sp.* TÜ10 and *Streptomyces sp*. TÜ102, were selected to activate silent clusters by applying the “semi-targeted” approach. According to internal data of the Tübingen natural products database, *S. sp.* TÜ17 is producing grisein, a sideromycin antibiotic active against Gram-negative and Gram-positive bacteria, already discovered in 1947 [53]. *S. sp*. TÜ10 is known as nonactin producer [54], whereas no biologically active metabolite has been isolated from *S. sp*. TÜ102 so far. 

Both plasmids pEM1 and pEM2 were introduced into the three strains, and the integration into their genomes was confirmed by PCR. 

#### 2.2.1. Metabolites Produced by Recombinant *Streptomyces sp*. TÜ17 Strains

**Figure 2:**
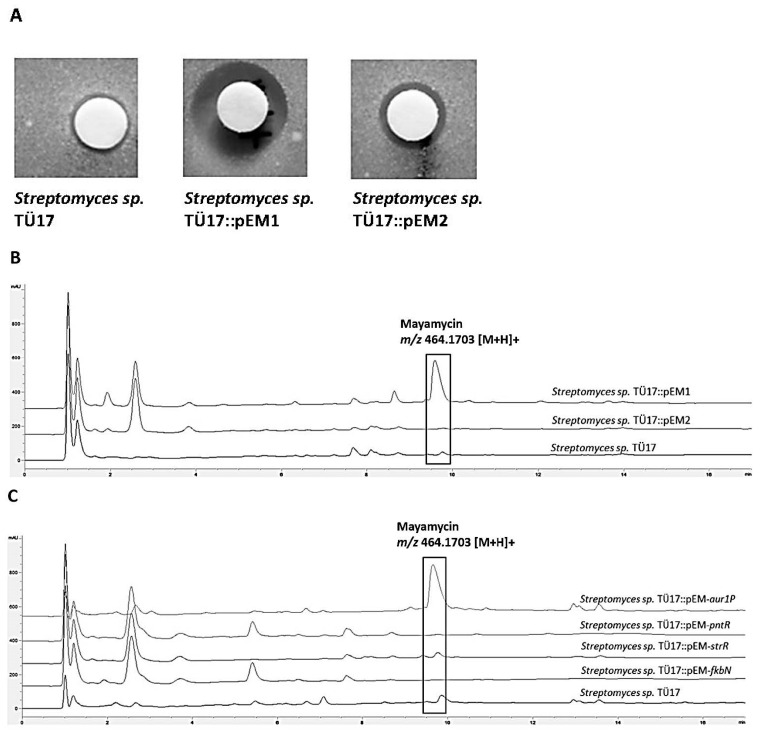
Analyses of the extracts of *S. sp.* TÜ17 wild type and recombinant strains. (**A**) Bioassay analysis of *S. sp.* TÜ17 and the recombinant strains *S. sp.* TÜ17::pEM1, *S. sp.* TÜ17::pEM2. Indicator strain *Bacillus subtilis*; 10 µL of the extracts were spotted of each culture on filter disc (1 mL ethylacetate extract was evaporated and dissolved in 100 µL methanol). Inhibition zone of *S. sp.* TÜ17::pEM1 ø 1.2 cm. (**B**) HPLC/MS analysis of *S. sp.* TÜ17 and the recombinant strains *S. sp.* TÜ17::pEM1, *S. sp.* TÜ17::pEM2. A peak with *m/z* 464.1703 [M+H]^+^, corresponding to mayamycin, was only detected in the extract of *S. sp.* TÜ17::pEM1. (**C**) HPLC/MS analysis of *S. sp.* TÜ17 and the recombinant strain *S. sp.* TÜ17::pEM-aur1P, *S. sp.* TÜ17::pEM-pntR, *S. sp.* TÜ17::pEM-strR, *S. sp.* TÜ17::pEM-fkbN, *S. sp.* TÜ17. The peak with *m/z* 464.1703 [M+H]^+^, corresponding to mayamycin, was only detected in the extract of *S. sp.* TÜ17::pEM-aur1P.

The integration of pEM1 in *S. sp*. TÜ17 resulted in activating at least of one BGC. Ethylacetate extracts of *S. sp*. TÜ17::pEM1 showed antibiotic activity against *B. subtilis* and *E. coli* in contrast to the recombinant strain *S. sp.* TÜ17::pEM2 and the wild type *S. sp.* TÜ17 (Figure 2A). The subsequent HPLC/MS analyses revealed a peak with a retention time (Rt) of 9.7 min and a molecular mass of *m/z* 464.3 [M+H]^+^. This peak was absent in both, *S. sp.* TÜ17 and *S. sp.* TÜ17::pEM2 extracts (Figure 2B). The molecular mass and the Rt of this peak did not match with data from the internal natural product database. To further characterise the compound, a high-resolution LC-MS analysis of the *S. sp.* TÜ17::pEM1 extract was performed. The identified mass *m/z* 464.1703 [M+H]^+^ was compared with the data of the molecules from the Dictionary of Natural Products (CRC Press, DNP 28.2). The masses were in accordance with the masses of mayamycin, an angucycline antibiotic also produced by a *Streptomyces* strain isolated from the marine sponge *Halichondria panicea* [55]. In addition, the MS/MS fragmentation pattern of the substance produced by *S. sp.* TÜ17::pEM1 also coincided with the pattern of the maymycin standard (SI. Figure S2) (kindly provided by J. Wiese, Helmholtz-Zentrum für Ozeanforschung Kiel, Germany). Mayamycin, a compound of the angucycline type, shows activity against several antibiotic-resistant strains, such as *Klebsiella pneumoniae*, *Pseudomonas aeruginosa*, *Staphylococcus aureus* and others, as well as against cancer cell lines, such as GXF251L (gastric cancer) LXF529L (non-small-cell lung cancer) MAXF401NL (mammary cancer) and many others [55].

Marine actinomycetes are still considered to be promising natural producers of new antibiotics with clinical relevance. They usually live associated with other organisms, such as sponges, but are difficult to isolate and cultivate. To activate the production of mayamycin, subinhibitory concentrations of the antibiotics bacitracin and tetracycline were added to the culture [55]. Applying our “semi-targeting” approach, we demonstrated that it is not necessary to use exotic sources to isolate interesting metabolites, but rather to exploit the potential of already isolated actinomycetes.

To confirm the specific activation of the cluster by the introduced regulators, we analysed the published mayamycin BGC, and the regulator gene *may18* contained therein [56]. Multiple sequence alignment of May18 and all CSRs encoded on plasmid pEM1 revealed the highest homology of May18 to Aur1P (SI. Figure S3). Moreover, BlastP analysis of Aur1P showed 70% identity and 81% similarity to May18. To verify the hypothesis that Aur1P indeed initiates activating the maymycin BGC, the individual CSR regulator genes were introduced into *S. sp*. TÜ17. The silent maymycin BGC could only be activated in the recombinant strain *Streptomyces sp*. TÜ17::pEM-*aur1P*, carrying the *may18* homologous gene (Figure 2C). From these results, it can be concluded that for activating BGC, a plasmid is needed that covers a broad spectrum of potential activator genes as comprehensively as possible. In our case, this was achieved by cloning CSR genes with high diversity.

#### 2.2.2. Metabolites Produced by Recombinant Strains *Streptomyces sp*. TÜ102 and *Streptomyces sp*. TÜ10 

After showing that the method generally works, we aimed to investigate the molecular mechanism of silent cluster activation by the introduced regulatory genes in more detail. Therefore, we selected two sequenced strains from the Tübingen strain collection. This has the advantage that in the case of successful activation of a silent BGC, the corresponding cluster could be identified more easily. We focused on a strain from which no compound has been isolated so far (*S. sp*. TÜ102, Accession Nr.: JAHDTK000000000) and on one from which several metabolites have already been characterised (*S. sp*. TÜ10, Accession Nr.: JAHDTJ000000000).

**Figure 3:**
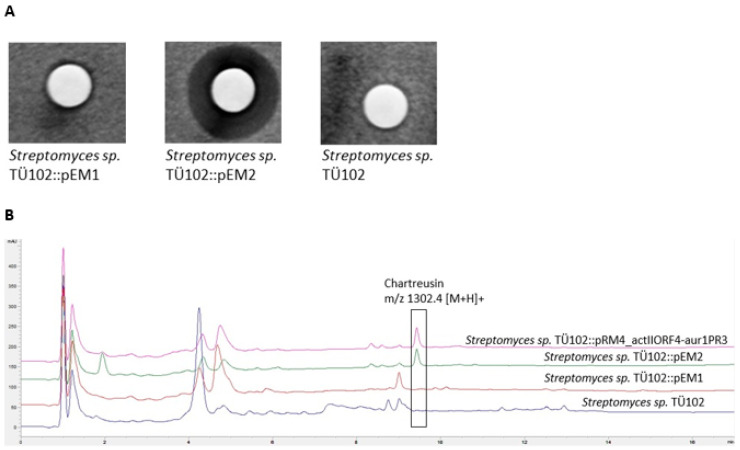
Analyses of the extracts of *S. sp.* TÜ102 wild type and recombinant strains. (**A**) Bioassay analysis of *S. sp.* TÜ102 and the recombinant strains *S. sp.* TÜ102::pEM1, *S. sp.* TÜ102::pEM2 and *S. sp.* TÜ102. Indicator strain *Bacillus subtilis;* 10 µL of the extracts were spotted of each culture on filter disc (1 mL ethylacetate extract was evaporated and dissolved in 100 µL methanol). Inhibition zone of *S. sp.* TÜ102::pEM2 ø 1 cm. (**B**) HPLC/MS analysis of *S. sp.* TÜ102 and the recombinant strains *S. sp.* TÜ102::pEM1, *S. sp.* TÜ102::pEM2, *S. sp.* TÜ102 and *S. sp.* TÜ102 pRM4_actIIORF4-aur1PR3. A peak with *m/z* 1302.4 [M+H]^+^, corresponding to chartreusin, was only detected in the recombinant strains *S. sp.* TÜ102::pEM2 and *S. sp.* TÜ102 pRM4_actIIORF4-aur1PR3.

Ethylacetate extracts of the recombinant strain *S. sp*. TÜ102::pEM2 showed antibacterial activity against *B. subtilis*, whereas no antibiotic activity was observed in extracts of the recombinant strain *S. sp.* TÜ102::pEM1 and the wild type *S. sp*. TÜ102 (Figure 3A). Thus, the introduction of the plasmid pEM2, carrying various SARP regulator genes, led to activating at least one silent cluster encoding the biosynthesis of a biologically active substance. HPLC-MS analyses of the extracts revealed a peak with a retention time (Rt) of 9.4 min and a molecular mass *m/z* 1302.4 [M+H]^+^, which could not be detected in the *S.* TÜ102::pEM1 and *S. sp*. TÜ102 extracts (Figure 3B). To evaluate which substance was produced, we compared the Rt and molecular mass of this peak with the data in our internal database. This matched with the data of chartreusin, a type II polyketide antibiotic (PK) with a mixed PK-carbohydrate structure. In addition to the antibiotic activities, chartreusin displays substantial chemotherapeutic activities [57]. Its mode of action (MoA) is based on the inhibition of topoisomerase II during DNA synthesis [58]. 

In the subsequent step, *in silico* analyses were performed to determine whether a potential chartreusin BGC is present in the genome of *S. sp*. TÜ102. For this reason, the genome sequence of *S. sp*. TÜ102 was evaluated by using the genome mining tool antiSMASH [7]. Thereby 34 potential BGC were predicted. One of them showed high similarities to the type II PKS BGC that codes for the synthesis of chartreusin. The chartreusin BGC has been identified and characterised in *Streptomyces chartreusis* HKI-249 [57]. The nucleotide sequence of the published chartreusin BGC (GenBank: MH540322.1) displays 99% nucleotide identity to the BGC in strain *S. sp.* TÜ102 (~35 kb of both BGCs were aligned). Both BGCs contain the same number of genes in an identical orientation. In addition to the structural genes responsible for the stepwise biosynthesis of the compound, both BGCs are coding for three regulatory genes: *chaR1*, encoding a putative transcriptional regulator of the SARP family, *chaR2*, encoding a putative sensor histidine kinase, and *chaR3*, encoding a putative transcriptional regulator of the LacI family [57]. Although both BGCs are nearly identical, the BGC in *S. sp.* TÜ102 remained silent under the cultivation conditions used; only the introduction of heterologous SARP regulatory genes in *S. sp.* TÜ102::pEM2 resulted in activating the BGC. 

For a detailed understanding of these findings, phylogenetic analyses were performed using the Phylogeny.fr tool. This involved comparison of the protein sequences of six SARPs encoded in the *Streptomyces sp*. TÜ102 genome and the protein sequences of SARPs introduced heterologously via plasmid pEM2. These analyses revealed the clustering of ActIIORF4 and Aur1PR3 with the chartreuse ChaR1-like SARP regulator (SI. Figure S4). The additional blastP analyses confirmed 51% identity of Aur1PR3 and 42% identity of ActIIORF4 with the ChaR1 SARP regulator. 

**Figure 4:**
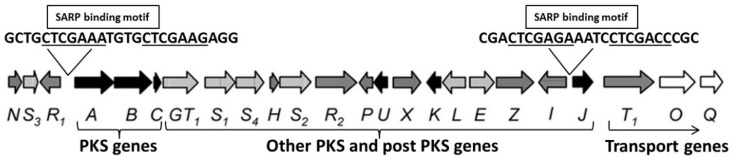
Gene organisation of the chartreusin biosynthetic gene cluster (BGC) (adapted from Xu et al., 2005). Two SARP binding motifs were identified within the chartreusin BGC. PKS, polyketide synthase.

Screening the promoter regions of the BGC genes for SARP binding motifs revealed two putative SARP binding motifs in chartreusin BGCs of both producer strains: The first (CTGCTCGAAATGTGCTCGAAGAGG) upstream of the *chaA-R* genes and the second (CGACTCGAGAAATCCTCGACCCGC) upstream of the *chaJ-Q* genes (Figure 4). Furthermore, the SARP binding site upstream of the *chaJ* gene was identical to the binding motif of the ActIIORF4 regulator within the Act BGC [40].

To subsequently specify which of the five introduced heterologous SARP regulatory genes was responsible for the activation, the regulatory genes were introduced individually into *S. sp*. TÜ102. Surprisingly, none of the individual regulatory genes was able to activate the putative chartreusin BGC. Consequently, we constructed the plasmid pRM4_actIIORF4-aur1PR3, bearing the genes *actIIORF4* and *aur1PR3*. Remarkably, the presence of these two SARPs, ActIIORF4 and Aur1PR3 in *S. sp.* TÜ102 resulted in the production of chartreusin, demonstrating that both regulators are required for activating the silent chartreusin BGC (Figure 3B). 

**Figure 5:**
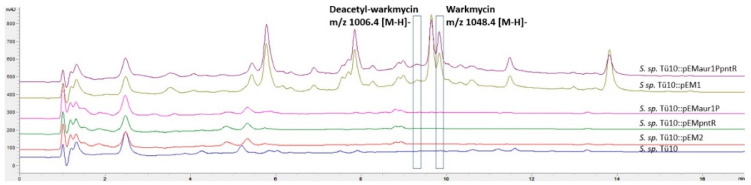
HPLC/MS analysis of the extracts of *S. sp.* TÜ10 wild type and recombinant strains. Extracts (1 mL ethylacetate extract was evaporated and dissolved in 100 µL methanol) of *S. sp.* TÜ10::pEMaur1P, *S. sp.* TÜ10::pEMpntR and *S. sp.* TÜ10::pEMaur1PpntR, *S. sp.* TÜ10::pEM1, *S. sp.* TÜ10::pEM2, *S. sp.* TÜ10 were analysed via HPLC/MS. A peak with *m/z* 1048.4 [M+H]^+^, corresponding to warkmycin and a peak with *m/z* 1006.4 [M+H]^+^ corresponding to deacetyl-warkmycin, were only detected in the recombinant strains *S. sp.* TÜ10::pEM1 and *S. sp.* TÜ10::pEMaur1PpntR.

In contrast, the second investigated strain *S. sp*. TÜ10 exhibited a different behaviour. Here, the ethylacetate extract of the wild-type strain *S. sp.* TÜ10 already revealed antibacterial activity against *B. subtilis*. In the HPLC/MS chromatograms of the extracts of the recombinant strain *S. sp*. TÜ10::pEM1, two additional peaks with an Rt of 9.2 min and a molecular mass *m/z* of 1006.4 [M+H] ^−^ and Rt 9.8 min and a molecular mass *m/z* 1048.4 [M+H]^−^, respectively, were present. Both peaks were missing in the extracts of the WT and the recombinant strain *S. sp.* TÜ10::pEM2 (Figure 5).

To determine the structural properties of the compounds associated with these peaks, the data were compared with the data of the internal natural product database. Both, the Rt and the masses corresponded to the already known natural products deacetyl-warkmycin (Rt 9.2 min, *m/z* 1006.4 [MH]^−^) and warkmycin (Rt 9.8 min, *m/z* 1048.4 [MH]^−^). Warkmycin is a glycosylated angucycline antibiotic with activity against gram-positive bacteria and tumour cell lines [59]. Warkmycin and deacetyl-warkmycin were extracted from *Streptomyces* strain Acta 2930 isolated from an embryonic sand dune in Warkworth Northumberland, UK [59]. Recently, two new members of the warkmycin family, warkmycin CS1 and CS2, were identified in a *Streptomyces* strain isolated from the integument of ants of the Tribe *Attini* [60]. To support the hypothesis that *S. sp*. TÜ10::pEM2 produced a warkmycin-like compound, we performed genome mining analyses to screen for a corresponding BGC. Using antiSMASH, 40 putative BGCs were identified. One of the predicted BGC showed a high similarity to the warkmycin cluster. It contains all structural genes described to encode the synthesis of warkmycin, as well as three genes coding for regulators (*warR1*, *warR2* and *warR3*). Blast analyses and multiple sequence alignment performed by Clustal Omega (SI. Figure S5) revealed similarities (56% identity, 73% positivity) of WarR1 to the Aur1P regulator, which acts as an activator of angucycline type auricin biosynthesis [61]. WarR2 belongs to the TetR family of regulators, and, like most regulators, in this family, could act as a repressor [60]. WarR3 is a putative regulator of the ArsR family and shows similarity (35% identity, 61% positivity) to PntR, a pentalenolactone BGC regulator [35]. Known representatives of this family are transcriptional repressors with an HTH domain that dissociates from DNA in the presence of metal ions (e.g., Zn^2+^, As^2+^, As^3+^, Sb^3+^, Cd^2+^) [62]. 

Again, we were interested in determining the regulator that is responsible for activating warkmycin. Therefore, the regulatory genes of the plasmid pEM1 were separately introduced into the strain *S. sp*. TÜ10. Based on the similarities of the two regulators encoded in the warkmycin cluster to the regulator genes *aur1P* and *pntR* that we introduced into *S. sp*. TÜ10, we hypothesised that both genes are necessary to activate the warkmycin cluster. For this reason, the plasmid pEMaur1P_pntR was constructed, including the corresponding regulator genes. In the recombinant strain *S. sp*. TÜ10::pEMaur1P_pntR the production of the warkmycin-like substance was again restored (Figure 5).

To further verify this result, we performed RT-PCR analyses. For this, we isolated RNA from the *S. sp* TÜ10 WT and the recombinant strains *S. sp* TÜ10::pEM1, *S. sp* TÜ10::pEMaur1P, *S. sp* TÜ10::pEMpntR and *S. sp* TÜ10::pEMaur1P_pntR after 72 h cultivation under production conditions. We demonstrated that the warkmycin genes *b9w61_07295* (annotated as beta-ACP synthase), *b9W61_07275* (annotated as cyclase) and *b9W61_07205* (annotated as glycosyl transferase) were only transcribed when both regulator genes *aur1P* and *pntR* were also transcribed. The presence of only one of the two regulatory genes, on the other hand, did not lead to the transcription of the biosynthetic genes. The regulator genes *warR1*, *warR2* and *warR3* were not transcribed, at all (SI. Figure S6). Based on these results, we concluded that only the presence of both regulatory genes *aur1P* and *pntR* under the control of the constitutive promoter ermEp* enabled activating the warkmycin cluster. 

## 3. Conclusions

We have developed a powerful tool to activate silent clusters in *Streptomyces* strains. By using a “semi-targeting” approach, metabolites are produced under standard cultivation conditions that are otherwise only synthesised by using specific techniques. These results indicate that new compounds can be identified when this approach is applied in an automated high-throughput procedure with many different strains. 

Our results illustrate the reasons why the induction of gene expression often fails: In many cases, multiple activators are needed. By introducing plasmids with a broad spectrum of regulator genes into the strains, we circumvent the problem and activate silent clusters that otherwise can only be activated using adapted methods.

## 4. Materials and Methods

### 4.1. Bacterial Strains and Cultivation Conditions

Different *Streptomyces* strains were used for the amplification of the regulator genes (Table 1).

*Streptomyces sp*. TÜ10, *Streptomyces sp*. TÜ17, *Streptomyces sp*. TÜ102, and *Streptomyces arenae* TÜ469 were selected from the Tübingen strain collection. *Escherichia coli* NovaBlue™ (Novagen, Germany, Darmstadt) was used for transformation; *E. coli* ET12567/pUZ8002 as non-methylating host for conjugation. *E. coli* K12 and *Bacillus subtilis* ATCC 6633 were used for bioassay tests.

*Streptomyces* strains were cultivated on SFM (20 g/L soy flour, 20 g/L mannitol, 10 mM MgCl_2_, 15 g/L agar) or HA (10 g/L malt extract, 4 g/L yeast extract, 4 g/L glucose, 1.46 g/L CaCl_2_, pH 7.3) solid medium at 28 °C for 5–10 days and in TSB (Tryptic soy broth—Difco Heidelberg, Germany) or R5 liquid medium (Kieser et al., 2000) by shaking (110 RPM) at 28 °C for 3–7 days. TSB liquid medium containing apramycin (50 μg/mL) and nalidixic acid (25 μg/mL) was used to cultivate the strains for DNA isolation or as 3-days-cultivation of preculture for inoculation into R5 liquid medium or HA solid medium to perform production tests. 

### 4.2. DNA Manipulations, Plasmids

All DNA manipulations in *E. coli* strains were done according to [63]. *Streptomyces* manipulations were done according to [64]. Restriction enzymes were used according to the recommendations of suppliers (Thermo Fisher Scientific, Waltham, MA, USA)

The integrative pRM4 vector [49] was used for cloning of regulatory genes under the control of the strong constitutive ermEp* promoter. The recombinant plasmids were integrated via *the* ΦC31 *att site* into the chromosome of the *Streptomyces* strains. 

### 4.3. Constructions of Plasmid pEM1 and pEM2 

Standard cloning techniques were used to construct plasmids pEM1 and pEM2. Primers for amplification are listed in Table S1. All cloned genes are under the control of the strong ermEp* promoter, and each gene carries RBS for its translation. All plasmids were isolated by QIAGEN Plasmid *Plus* Midi Kit (Qiagen, Hilden, Germany) and sequenced.

Genomic DNA (gDNA): Genomic DNA from *Streptomyces* strains was isolated using the VWR peqGOLD Bacterial DNA kit (VWR international GmbH, Pennsylvania, USA). PCR with gDNA as a template was used to confirm the integration of plasmids. 

### 4.4. Phylogenetic Analysis 

Phylogenetic analysis was performed by Phylogeny.fr using MUSCLE Alignment, Gblocks Curation, PhyML+ aLTR Phylogeny and TreeDyn Tree Rendering. http://www.phylogeny.fr/ (7 April 2020) [65].

### 4.5. Multiple Sequence Alignment 

An omega cluster tool was used to perform multiple sequence alignments with standard settings using HMM profile-profile techniques [66].

### 4.6. Conjugation 

To introduce the plasmids into different *Streptomyces* strains, non-methylating *E. coli* ET12567/pUZ8002 competent cells were transformed with each plasmid and selected on plates containing apramycin (50 μg/mL), chloramphenicol (25 μg/mL) and kanamycin (50 μg/mL). *E. coli* ET12567/pUZ8002 is resistant against chloramphenicol and is carrying a pUZ8002 plasmid containing genes needed for DNA transfer and kanamycin resistance. *E. coli* ET12567/pUZ8002 with plasmids pEM1 or pEM2 were grown to OD = 0.6 in liquid LB medium with apramycin (50 μg/mL), chloramphenicol (25 μg/mL) and kanamycin (50 μg/mL). The *E. coli* cells were washed with fresh LB, while *Streptomyces* spores (10^8^) were heat-shocked at 50 °C for 10 min. Spores and cells were mixed and plated on an SFM medium containing 10 mM MgCl_2_. After 16h, plates were overlayed with 20 μL nalidixic acid (25 mg/mL) and 25 μL apramycin (50 mg/mL) and cultivated at 30 °C for 4–7 days. 

For conjugation, spores of *Streptomyces* were harvested from the SFM or HA medium, and SFM medium was used to plate mating. 

### 4.7. Sample Collection and Extraction

Both plasmids pEM1 and pEM2 were introduced into the three strains, respectively, and the integration into their genomes was confirmed by PCR. Using the recombinant strains *Streptomyces sp.* TÜ17::pEM1, *Streptomyces sp.* TÜ17::pEM2, *Streptomyces sp*. TÜ102::pEM1, *Streptomyces sp*. TÜ102::pEM2, *Streptomyces sp*. TÜ10::pEM1 and *Streptomyces sp*. TÜ10::pEM2 production experiments were performed. Strains *Streptomyces sp.* TÜ17, *Streptomyces sp.* TÜ17::pEM1, *Streptomyces sp.* TÜ17::pEM2, *Streptomyces sp*. TÜ102, *Streptomyces sp*. TÜ102::pEM1, *Streptomyces sp*. TÜ102::pEM2 were grown as a preculture in TSB medium (containing apramycin (50 μg/mL) and nalidixic acid (25 μg/mL)) and inoculated for the production test into 50 mL R5 media. Strains *Streptomyces sp*. TÜ10, *Streptomyces sp*. TÜ10::pEM1 and *Streptomyces sp*. TÜ10::pEM2 were grown as a preculture in TSB medium (containing apramycin (50 μg/mL) and nalidixic acid (25 μg/mL)) and inoculated for the production test into 50 mL HA media. The recombinant strains and the corresponding wild type (WT) strains were cultivated in three independent cultures for 7 days. Samples (1 mL) were taken after 3, 5 and 7 days and were extracted with equal amounts of butanol or ethylacetate. Extracts were evaporated in an Eppendorf concentrator 5301 until dry and dissolved in 100 µL of 100% methanol. 

### 4.8. Bioassay

Extracts of the recombinant strains and the corresponding WT strains were spotted on antibiotic discs and tested against *B. subtilis* ATCC 6633 and *E. coli* K12. Reference strains (*B. subtilis* or *E. coli*) were grown in liquid LB medium to OD = 0.6 and inoculated into agar plates (OD = 0.2). Plates were stored open for drying for 20 min under the sterile bench. The spotted antibiotic discs were placed on plates. Strains were incubated overnight by 28 °C or 37 °C. 

### 4.9. HPLC-MS/MS Analysis

The HPLC-MS-System: Agilent HPLC-MS-system consisting of: 1200 binary pump, 1200 automatic sampler, thermostated, 1200 column thermostate, 1200 diode array detector with 10 mm standard flowcell, LC/MSD Ultra Trap System XCT 6330 (Agilent, Waldbronn).

HPLC parameters: stationary phase: Nucleosil 100 C18 3 µm, 100 × 2 mm ID with a precolumn 10 × 2 mm ID (Dr. Maisch GmbH, Ammerbuch), column temperature: 40 °C, mobile Phase: A = 0.1% formic acid, B = 0.06% formic acid in acetonitrile, gradient: t_0_ = 10% B, t_15_ = t_17_ = 100% B, posttime 6 min. 10% B, flowrate: 400 µL/min, injection volume: 2.5 µL, detections wavelengths (bandwidth): 230 nm (10 nm), 260 nm (20 nm), 280 nm (20 nm), 360 nm (20 nm), 435 nm (40 nm), software: LC/MSD ChemStation Rev. B.01.03, Agilent.

MS parameters: ionisation: ESI (positive and negative, alternating), mode: ultra-scan, capillary voltage: 3.5 kV, temperature: 350 °C, target mass: *m*/*z* 800, software: 6300 series trap control version 6.1, Bruker Daltonik (Agilent, Waldbronn, Germany).

## Figures and Tables

**Table 1:** Sources for the amplification of regulatory genes.

Strain	Regulatory Genes
*Streptomyces griseus* DSM 40236	*griR*, *strR*
*Streptomyces coelicolor* M145	*actIIORF4*, *redD*
*Streptomyces tsukubaensis*	*fkbN*
*Streptomyces arenae* TÜ469	*pntR*
*Streptomyces pristinaespiralis*	*papR2*
Cosmid pCos51 (Novakova et al., 2005)	*aur1P* and *aur1PR3*

## Data Availability

Not applicable.

## Supplementary Materials

The following are available online at https://www.mdpi.com/article/10.3390/ijms22147567/s1.
