# Supplementary material for: A Regulator Based “Semi-Targeted” Approach to Activate Silent Biosynthetic Gene Clusters
Source: Int J Mol Sci. 2021 Jul 15;22(14):7567. doi: 10.3390/ijms22147567 (PMC8306873; doi:10.3390/ijms22147567)
Supplement: Supplementary file 1 [file ijms-1281596-SI.pdf › ijms-1281596-SI.pdf]

Supplementary

Table S1: Primers for plasmids construction and integration confirmation

Primers for gene amplification	
CSRs	
aur1PFwNde	CCCCATATGAACCAGCGGACCATGGCTGTG
aur1PRevHind	TTTTAAGCTTTCAGCCCTGGCCGAAGCGGAATC
PntRFwHind	TTTTAAGCTTCTACCGTGCGGCTTCGGCTC
PntRRevNde	TTTTCATATGGTGACTCCTACTCAGCGTGCC
strRFwNde	CCCCCATATGGAGCATATTTCAGGGAACAGCC
strRRevHind	CCCCAAGCTTTCATCCGACATCGCTCAAGG
FkbNFwNde	AAAACATATGGTGGTTCCGGAAGTGCGAGC
FkbNRevHind	AATAAGCTTCTACCCGCACCGGTCGCCC
SARPs	
actFwNde	GGGGGGGCATATGAGATTCAACTTATTGGGACG
actRevHind	CCCCAAGCTTCTACACGAGCACCTTCTCAC
griRFwNde	CCCCCCATATGTCTACGCACCATCAATTCTC
griRRevHind	TTTTAAGCTTTCAGCCGGAGCGCCCTTTCG
redDFwNde	TTTTCATATGTCAGGCGCTGAGCAGGCTGGTG
redDRevHind	AAAAAAGCTTATGACGGGTGGGGGAGTGCTTG
papR2FwNde	GGGGGCATATGAAGTTCCGCATTCTCGGTC
papR2RevHind	TTTTAAGCTTCTAGTGGCCCGAGGCCGGGTTG
aur1PR3FwNde	CCCCATATGGTGGACATCGATGTACTG
aur1PR3RevHind	TTTTAAGCTTTCAGCCCACTCGGGCGAGCCGCTTG
Next step primers CSRs	
Fwpnt_RBS_REcoNotBln	GGGAATTCGCGGCCGCCCTAGGAATCCTACTAAGTGACTCCTACTCAGCGTGC
Revpnt_RXbaMfeBsrG	TTTCTAGACAATTGTGTACACTACCGTGCGGCTTCGGCTC
strR_RBS_FW_Not	TTATCCTGAATTCTCCTCCGCGGCCGCATGGAGC
strR_Rev_BlnI	TCTAATCCTAGGTCATCCGACATCGC
fkbN_RBS_Fw_BsrGI	TGGCAATGTACAGTCAATTCCTCCGTGGTTCCGGAAGTGC
fkbN_Rev_MfeI	GCTAGGACAATTGCTACCCGCACCGGT
Next step primers SARPs	
griRFw_RBS_HindIII	GGGGAAGCTTACTCCTCGATGCTAGTCGCGGTTGAT
griRRevXbaNotAflBsrGMfeKpnI	TCATTCTAGAGCGGCCGCCTTAAGTGTACACAATTGGGTACCAGCTTTCAGCCGGAGCGCCCTTTC
aur1PR3_RBS_Fw_KpnI	CAGCAGGGTACCTATTCCTCCATGGTGGACATCGATG
aur1PR3_Rev_MfeI	CAGCAGCAATTGTCAGCCCACTCGGGCG
papR2_RBS_Fw_BsrG	CAATTGTCCTCCTGTACAATGAAGTTCCGC
papR2_Rev_AflII	CATCATCTTAAGCTAGTGGCCCGAGG
redD_RBS_Fw_NotI	CATCATCTTGCGGCCGCAAGTCCTCCATGACGGGTG
redD_Rev_XbaI	CAGCAGTCTAGAAGGCCTTCAGGCGCTG
Strain confirmation	
pRM4SeqFw	CACACGTGGCACCGCGATGCTGTTG
ApraFw	GGCATCGCATTCTTCG
ApraRev	CATGTGCAGCTCCATCAG

RT-PCR	
RT1Fw	CGGAACGAAGGCGTTCTGGGAAC
RT1Rev	CTCCTCGTCGAGGCTCATGGTG
RT2Fw	ACTTCATCGACGAGGCCCAGCTC
RT2Rev	TGTGCTGGGACGTCGCCACGAC
RT3Fw	CGTCGGCACTCCCCGGATCC
RT3Rev	CGTGGTAGGCGAGGGTCCGCC
hrdA_WAR_Rev	GCGAGGCGGGAGTCGAGGTC
hrdA_WAR_Fw	GAACAGGTCGCTGCACCACC
aur1P_RT_Fw	GAAGCGGAATCCGACGCCG
aur1P_RT_Rev	CACCATCACCTGCGGGCCAC
pntR_RT_Fw	TACCGTGCGGCTTCGGCTCC
pntR_RT_Rev	CCTGGCGAGTTCGGTGAGC
Reg1fwWAR	GCTTCCCCCTGATCGAGCTGG
Reg1RevWAR	GAATCAATGCGGTAATGCGACGC
Reg2FwWAR	TACTCCGCTTCCCTTGTCCGCTTC
Reg2RevWAR	GAGATTCTGGTGCTGCTGCGGG

A

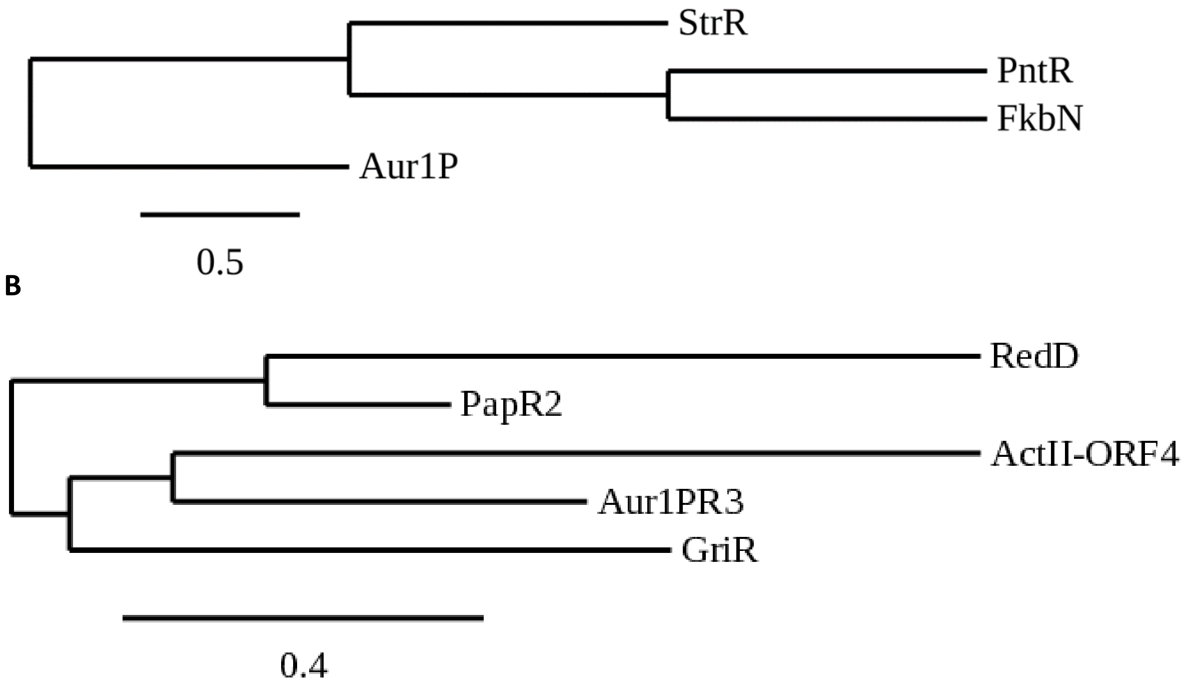

SI. Figure S1B: Phylogenetic analysis (Clustal Omega) of different regulators

(A) CSRs encoded on plasmid pEM1

(B) SARPs encoded on plasmid pEM2.

The bar below indicates the scale of branch lengths, representing the number of genetic alterations.

A

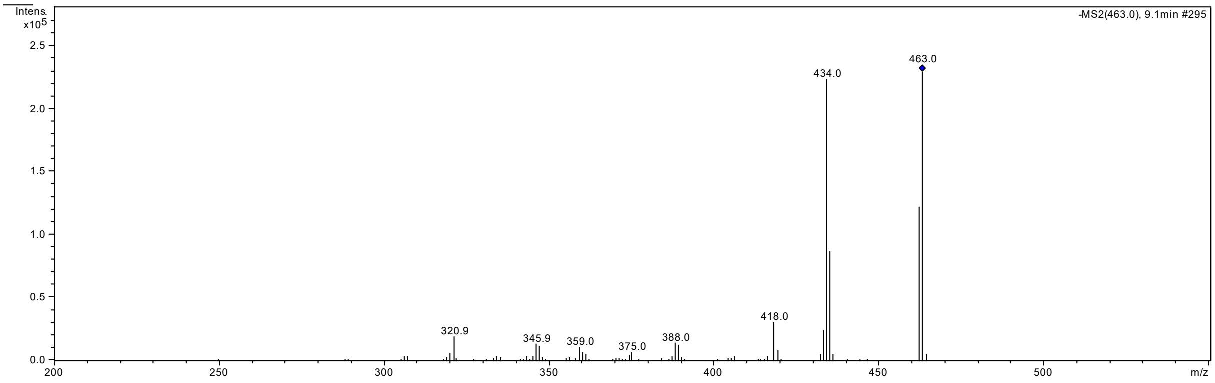

B

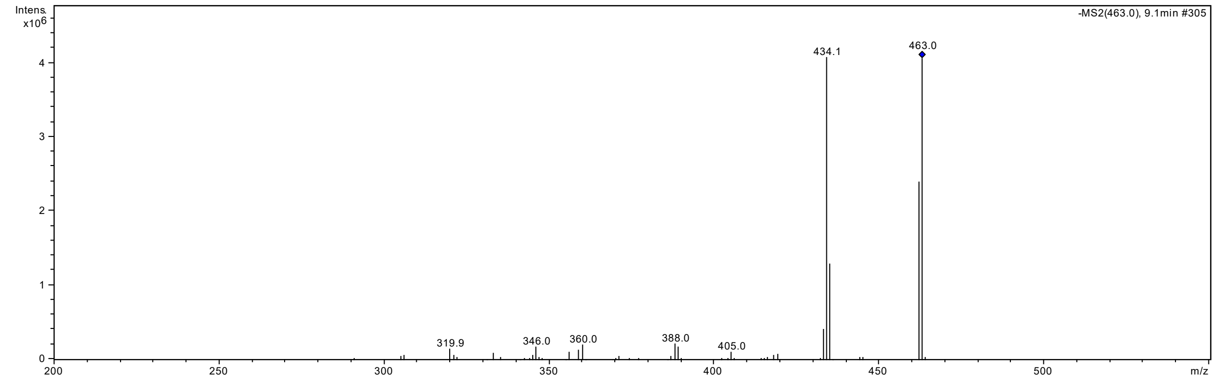

SI. Figure S2: MS/MS fragmentation of mayamycin

(A) Mayamycin standard (kindly provided by J. Wiese, Helmholtz-Zentrum für Ozeanforschung Kiel)

(B) Mayamycin isolated from *Streptomyces* sp. Tü17.

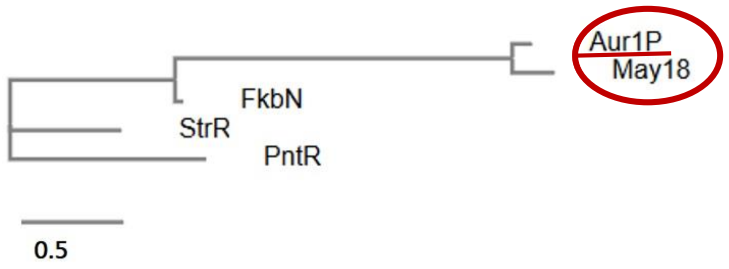

SI. Figure S3: Guided tree of the May18 and CSRs

Guided tree (Clustal Omega) of the May18 mayamycin BGC regulator and CSRs encoded on pEM1, based on multiple sequence alignment.

The bar below indicates the scale of branch lengths, representing the number of genetic alterations.

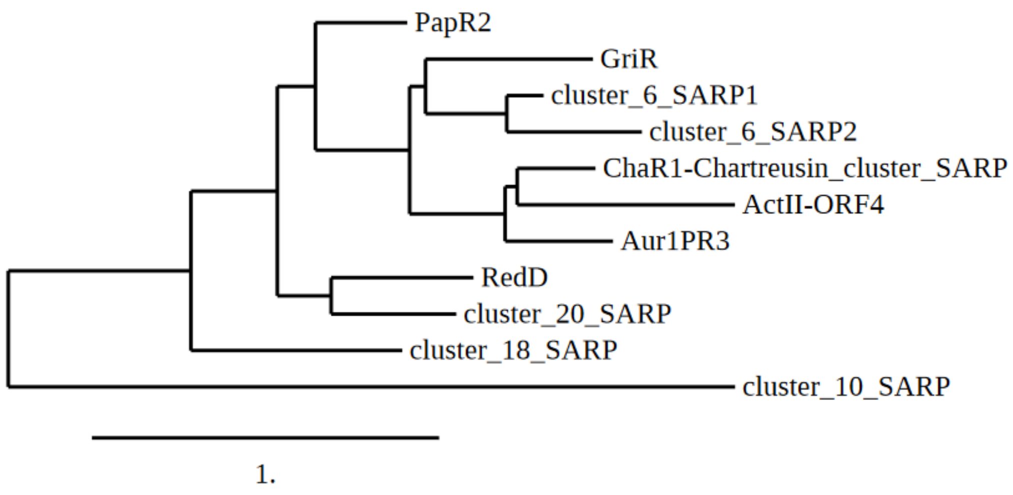

SI. Figure S4: Phylogenetic analysis (Phylogeny.fr) of SARPs encoded in the genome of *Streptomyces sp.* TÜ102 and on pEM2 plasmid.

The SARPs encoded in the genome of S. sp TÜ102 used for the phylogenetic analysis were identified within the clusters predicted by AntiSMASH (Blin el al., 2019). In one of the clusters (cluster 6), two SARP regulators (SARP1 and 2) are encoded

The bar below indicates the branch scale of the phylogenetic tree alterations.

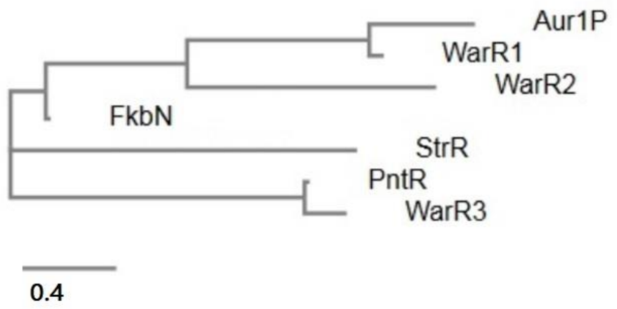

SI. Figure S5: Guided tree of the WarR-regulators and CSRs

Guided tree (Clustal Omega) of three identified warkmycin regulators WarR1, WarR2 and WarR3 and CSRs encoded on pEM1, based on multiple sequence alignment.

The bar below indicates the scale of branch lengths, representing the number of genetic alterations.

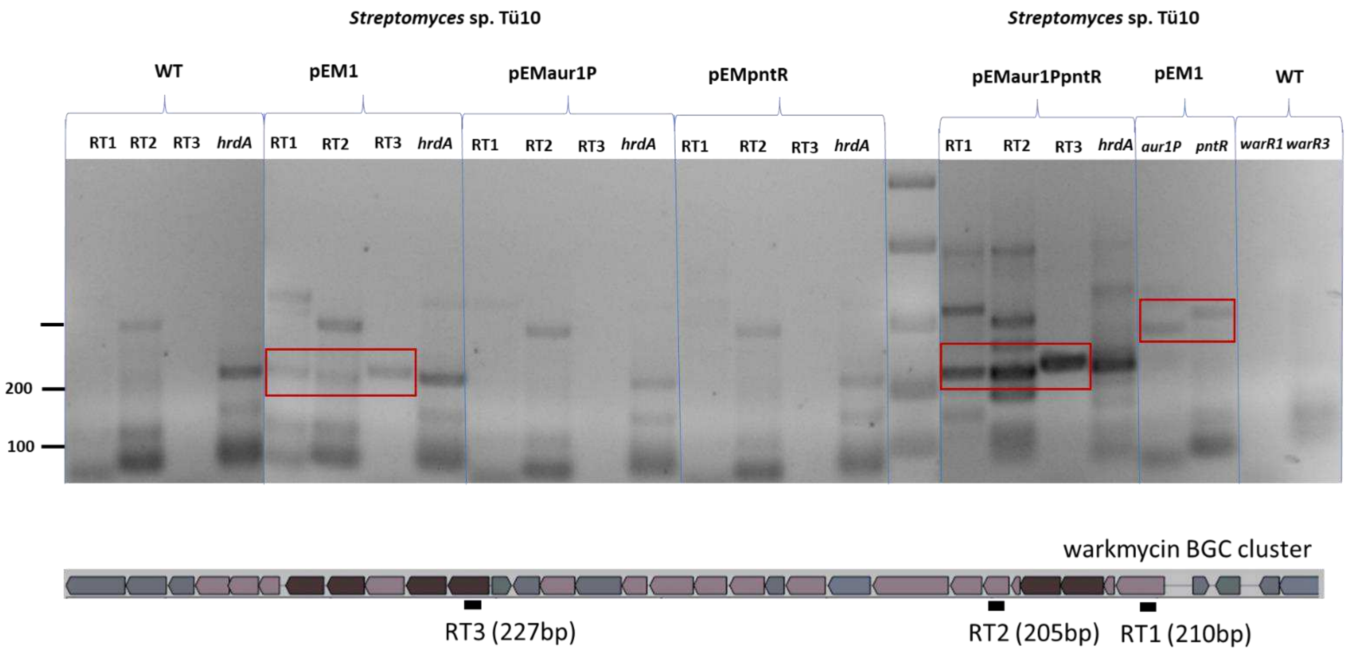

SI. Figure S6: RT-PCR analysis of gene expression in *S. sp* TÜ10 wild type (WT) and recombinant strains

Total RNA was prepared from *S. sp*. TÜ10 WT and the recombinant strains *S. sp* TÜ10::pEM1, *S. sp* TÜ10::pEMaur1P, *S. sp* TÜ10::pEMpntR and *S. sp* TÜ10::pEMaur1P_pntR after 72 h of cultivation. Fragments (RT1, RT2 and RT3) of the three structural genes and the regulator genes *warR1*, *warR2* *aur1P* and *pntR* were selected and evaluated as examples. The *hrdA* gene was used as control for RT-PCR.

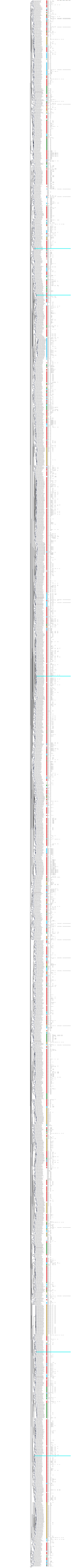
Streptomycetales
Micromonosporales
Pseudonocardiales
Corynebacteriales
Other
